# Relationship of Late Lactation Milk Somatic Cell Count and Cathelicidin with Intramammary Infection in Small Ruminants

**DOI:** 10.3390/pathogens9010037

**Published:** 2020-01-01

**Authors:** Giulia Maria Grazia Puggioni, Vittorio Tedde, Sergio Uzzau, Simone Dore, Manuele Liciardi, Eugenia Agnese Cannas, Claudia Pollera, Paolo Moroni, Valerio Bronzo, Maria Filippa Addis

**Affiliations:** 1 Porto Conte Ricerche, 07041 Alghero, Italy; 2 Department of Biomedical Sciences, University of Sassari, Via Vienna 2, 07100 Sassari, Italy; 3 Mediterranean Center for Disease Control, Via Vienna 2, 07100 Sassari, Italy; 4 National Reference Center for Sheep and Goat Mastitis, Istituto Zooprofilattico Sperimentale della Sardegna, Via Duca degli Abruzzi, 07100 Sassari, Italy; 5 Department of Veterinary Medicine, University of Milan, Via dell’Università 6, 26900 Lodi, Italy; 6 Quality Milk Production Services, Cornell University, 240 Farrier Rd, Ithaca, NY 14853, USA

**Keywords:** subclinical mastitis, sheep, goat, late lactation, cathelicidin ELISA, somatic cell count, bacteriological culture

## Abstract

Late lactation is a critical moment for making mastitis management decisions, but in small ruminants the reliability of diagnostic tests is typically lower at this stage. We evaluated somatic cell counts (SCC) and cathelicidins (CATH) in late lactation sheep and goat milk for their relationship with intramammary infections (IMI), as diagnosed by bacteriological culture (BC). A total of 315 sheep and 223 goat half-udder milk samples collected in the last month of lactation were included in the study. IMI prevalence was 10.79% and 15.25%, respectively, and non-aureus staphylococci were the most common finding. Taking BC as a reference, the diagnostic performance of SCC and CATH was quite different in the two species. In sheep, receiver operating characteristic (ROC) analysis produced a higher area under the curve (AUC) value for CATH than SCC (0.9041 versus 0.8829, respectively). Accordingly, CATH demonstrated a higher specificity than SCC (82.92% versus 73.67%, respectively) at comparable sensitivity (91.18%). Therefore, CATH showed a markedly superior diagnostic performance than SCC in late lactation sheep milk. In goats, AUC was <0.67 for both parameters, and CATH was less specific than SCC (61.90% versus 65.08%) at comparable sensitivity (64.71%). Therefore, both CATH and SCC performed poorly in late lactation goats. In conclusion, sheep can be screened for mastitis at the end of lactation, while goats should preferably be tested at peak lactation. In late lactation sheep, CATH should be preferred over SCC for its higher specificity, but careful cost/benefit evaluations will have to be made.

## 1. Introduction

Mastitis due to intramammary infection (IMI) [1,2] is one of the most significant health problems in dairy goat and sheep farming. Modern management strategies and udder health monitoring plans have contributed to lessening its impact, but milk yield and quality reductions due to mastitis are still responsible for relevant economic losses in both dairy species [3,4,5,6,7]. Therefore, the availability of diagnostic tools enabling a reliable assessment of udder health is of outstanding importance for maintaining farm profitability. Small ruminants with reduced productions due to subclinical IMI are typically replaced around the drying period based on the milk somatic cell count (SCC), usually assessed with the California mastitis test (CMT) [8,9]. The SCC value lies in the notion that udder invasion by pathogenic bacteria (an intramammary infection, IMI) triggers an inflammatory reaction (mastitis), with a consequent influx of leukocytes in the milk, mainly represented by polymorphonuclear neutrophil granulocytes, leading to an increase in its total cell concentration [10]. In small ruminants, however, and especially in goats, the SCC is influenced also by numerous non-infectious factors, including the stage of lactation. This is one of the major contributors to its decreased diagnostic value before the drying period, a moment in which reliable assessment of udder health is of great importance [11,12,13,14,15]. Bacteriological culture of milk (BC) is still the best way for detecting contagious mastitis outbreaks and for gaining a detailed understanding of the causes and dynamics of subclinical mastitis (SCM) events in the herd or flock [3,16]. Its applicability to routine screening, however, is limited by cost and sensitivity issues [7,17,18,19].

Among other useful protein markers, innate immune mediators and effectors, such as haptoglobin, Serum Amyloid A (SAA), and cathelicidins (CATHs) increase significantly in milk upon infection [20,21]. Based on shotgun proteomics, CATHs are among the most increased proteins in the milk of sheep with bacterial mastitis [22,23,24] and in the milk of goats with experimentally-induced endotoxin mastitis [25]. CATHs are antimicrobial proteins released by neutrophils and mammary epithelial cells upon infection [21,22,23,26,27], and may therefore represent reliable markers of mammary gland inflammation. With the aim of improving the sensitivity and specificity of mastitis detection, we developed a CATH ELISA, building on the sheep gene sequences. The assay showed good diagnostic performances in comparison to SCC and BC in mid-lactation ewes and cows [28,29]. The CATH assay was also demonstrated to be suitable for the detection of cathelicidins in goat milk when physiological levels were assessed over a full lactation year in comparison to the SCC [12]. That study confirmed the significant increase in both markers along the course of lactation in the milk from healthy goat udders, and revealed that CATH increased less than SCC. With these premises, we carried out a comparative study of SCC and CATH levels on both species in late lactation milk, to investigate their ability to detect mastitis due to subclinical IMI in this critical moment.

## 2. Results

### 2.1. Prevalence of Intramammary Infection According to Milk Bacteriological Culture Results

According to BC, IMI prevalence was 10.79% in sheep (34 out of 315) and 15.25% in goats (34 out of 223), with non-aureus staphylococci being the most prevalent microorganisms in both small ruminant species (52.94% in sheep and 85.29% in goats). In sheep, the remaining 16 samples were positive to *Enterococcus faecalis* (29.41%), *Streptococcus uberis* (11.76%), and *Klebsiella* spp. (5.89%). In goats, the remaining five samples were positive to *Staphylococcus aureus* (11.76%) and *Streptococcus dysgalactiae* (2.94%).

### 2.2. Relationship of Somatic Cell Counts and Cathelicidin Levels with Bacteriological Culture Results

The distribution of SCC and CATH levels in late lactation milk without or with IMI was assessed in both small ruminant species by taking BC results as a reference.

#### 2.2.1. Sheep

**Figure 1:**
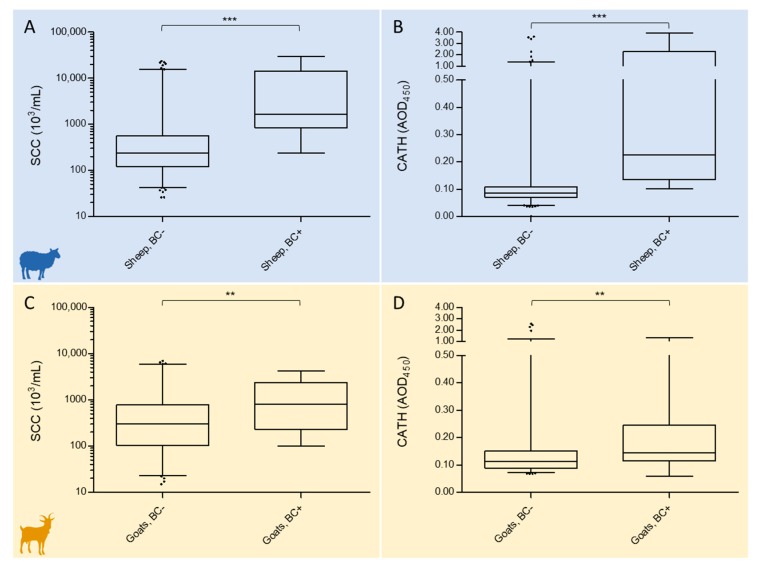
Boxplots illustrating the distribution of somatic cell count (SCC) and cathelicidin (CATH) values according to the bacteriological culture (BC) result in late lactation sheep (**A, B**) and goat milk (**C, D**). Boxes indicate values within the 25th and 75th percentiles, and the central line indicates the median value. Whiskers indicate values within the 2.5th and 97.5th percentiles, and individual dots represent values outside the whiskers. Median and IQR values are detailed in Table 1. The difference between classes within the same species was statistically significant (***p ≤ 0.0001; **p ≤ 0.005).

In sheep, BC-negative milk had significantly different levels (*p* < 0.0001) of both SCC and CATH than BC-positive milk. Value distributions of SCC and CATH in sheep milk are illustrated in Figure 1A,B, respectively, while median and interquartile range (IQR) values are detailed in Table 1.

#### 2.2.2. Goats

In goats, BC-negative and BC-positive milk also showed different SCC and CATH levels, but the difference was less significant *(p* < 0.005) and less pronounced than in sheep. Value distributions of SCC and CATH in goat milk are illustrated in Figure 1C,D, respectively, while median and IQR values are detailed in Table 1.

### 2.3. Test Characteristics of Somatic Cell Count and Cathelicidin Levels Based on Bacteriological Culture Results

**Figure 2:**
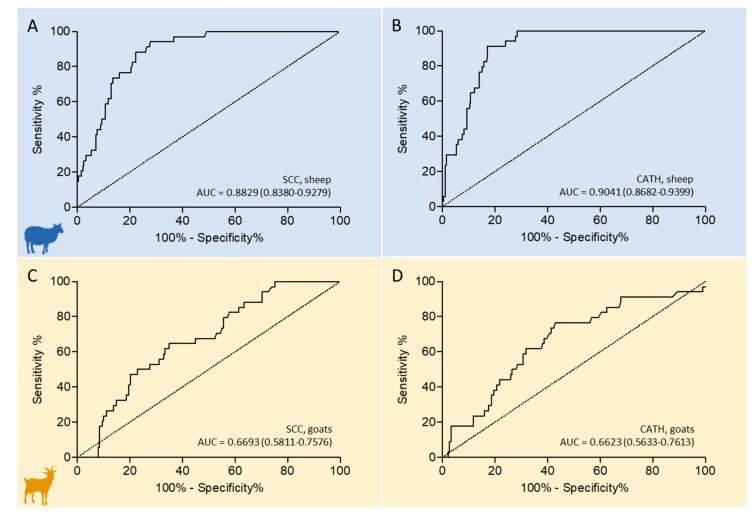
Receiver operating characteristic (ROC) curves illustrating the relationship of the somatic cell count (SCC, **A** and **C**) and cathelicidins (CATH, **B** and **D**) in late lactation sheep (top) and goat milk (bottom), with bacteriological culture results as the reference. ROC curves describe the tradeoff between sensitivity and specificity. The 45° diagonal of the ROC space is the random chance line. The respective area under the curve (AUC) values and their 95% confidence intervals are reported in the plot for each curve.

For assessing the respective test characteristics in late lactation milk of both species, receiver operating characteristic (ROC) curves were generated for SCC and CATH by using BC results as the reference, and area under the curve (AUC), optimal cut-point (c*), Youden index (J), sensitivity (Se), and specificity (Sp) were calculated. Results are plotted in Figure 2 and detailed in Table 2. Supplementary File 1 reports the results for each sample, while Supplementary Files 2 and 3 provide a schematic, comparative overview of the results obtained with the different tests (SCC, CATH, BC).

#### 2.3.1. Sheep

ROC curves revealed a good diagnostic performance of both SCC and CATH in late lactation sheep milk (Figure 2, left), with a higher AUC for CATH (0.9041, Figure 2B) than SCC (0.8829, Figure 2A). In sheep, the J values associated with the best Se/Sp tradeoffs were observed at a c* of 488.5 × 10^3^ cells/mL for SCC and of 0.1206 A0D450 for CATH, respectively. At c* values, SCC Se% was higher than CATH Se% by 2.94 percentage points (94.12 versus 91.18, respectively), but CATH showed a remarkably higher Sp%, by 10.68 percentage points (72.24 versus 82.92, respectively), as detailed in Table 2. At comparable Se% levels of 91.18 (obtained with an SCC threshold of 518.5 cells 10^3^/mL), CATH Sp% was 82.98 and SCC Sp% was 73.67. Therefore, CATH Sp% was 9.25 percentage points higher than SCC Sp%.

#### 2.3.2. Goats

On the other hand, the ROC curves of SCC and CATH revealed a poor diagnostic performance of both parameters in late lactation goat milk (Figure 2, right). In this species, the AUC of SCC (0.6693, Figure 2C) was slightly higher than the AUC of CATH (0.6623, Figure 2D). In goats, J values associated with the best Se/Sp tradeoffs were at a c* of 422 × 10^3^ cells/mL for SCC and of 0.1183 A0D450 for CATH, respectively. At these c* values, CATH Se% was higher than SCC Se% by 5.88 percentage points (Table 2), but Sp% was almost equally lower. CATH Sp% was 3.18 percentage points lower (61.90) than SCC Sp% (65.08) at comparable Se% levels (64.71), obtained with a threshold of 0.1265 Adjusted Optical Density at 450 nm (AOD450).

### 2.4. Relationship of Cathelicidin ELISA Results with the Somatic Cell Count

The relationship between CATH and SCC in late lactation sheep and goat milk was then evaluated by using the c* values defined by the ROC curve analysis as CATH ELISA thresholds.

#### 2.4.1. Sheep

**Figure 3:**
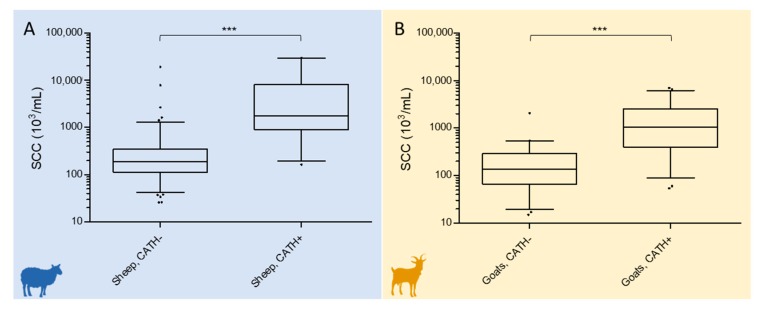
Log_10_ distribution of the SCC (cells × 10^3^/mL) in CATH-negative and CATH-positive late lactation sheep (**A**) and goat milk (**B**). Boxes indicate values within the 25th and 75th percentiles, and the central line indicates the median value. Whiskers indicate values within the 2.5th and 97.5th percentiles, and individual dots represent values outside the whiskers. The difference between CATH-negative and CATH-positive SCC classes was statistically significant in both species (*** P ≤ 0.0001).

CATH-negative (236, 74.92%) and CATH-positive (79, 25.08%) milk had significantly different SCCs *(p* < 0.0001). Median and interquartile range (IQR) SCC × 10^3^ were 191.5 (112.3–342.0) for CATH-negative and 1770 (911.0–8030.0) for CATH-positive sheep milk, respectively. The distribution of SCC values is plotted in Figure 3A.

#### 2.4.2. Goats

CATH-negative (116, 52.02%) and CATH-positive samples (107, 47.98%) had significantly different SCCs *(p* < 0.0001) also in this species. Median and interquartile range (IQR) SCC × 10^3^ were 137.5 (62.25–292) for CATH-negative and 1038 (400.0–2548) for CATH-positive goat milk, respectively. The distribution of SCC values is plotted in Figure 3B.

## 3. Discussion

Late lactation and the drying period are critical moments for making herd management decisions. At this time, however, the diagnostic specificity of the most widespread mastitis assay, the milk SCC, is known to be affected also by non-infectious factors. A better understanding of SCC behavior and reliability is therefore needed for its successful application, accompanied by the discovery and implementation of alternative markers and diagnostic strategies. Here, we evaluated a CATH ELISA in comparison to the milk SCC for their diagnostic performance in detecting small ruminant infectious mastitis, as diagnosed by BC in late lactation.

As a result, SCC and CATH levels were significantly different in BC-negative and BC-positive milk of both species; however, the difference between classes was more significant in sheep than in goat milk. The median values of both SCC and CATH were lower in BC-negative sheep milk than in BC-negative goat milk; on the other hand, the SCC of BC-positive milk was higher in sheep, leading to a better separation of BC-negative and BC-positive samples in sheep than in goats. The same was observed for CATH: the levels measured in uninfected and infected milk were quite different in sheep, while there was a more pronounced overlap of the two sample classes in goats. Accordingly, the median (IQR) values of CATH in uninfected goats were higher than in uninfected ewes, reducing the distance between the upper IQR value of uninfected milk and the lower IQR value of infected milk, and thereby compromising diagnostic performance. This occurred also if the median SCC value of uninfected goat milk was lower than in our previous study on Alpine goats, where we observed SCC > 4000 × 10^3^ and CATH > 0.2 AOD450. Breed is known to influence the SCC [30]; two-thirds of the goats in the present study were Saanen, and the higher amount of milk produced by this breed might account for these differences.

This was reflected in the remarkable differences between SCC and CATH for mastitis detection performance in the milk of the two species. In ewes, SCC reliability was acceptable, but CATH showed superior test characteristics, especially in terms of specificity. In goats, neither SCC nor CATH provided satisfactory results: AUC, Se%, and Sp% values were low for both markers and were in line with those observed in our previous study along the course of an entire lactation [12].

The dramatically different performance of the CATH ELISA in the two species might be related to their different milk cell type composition. Although produced also by mammary epithelial cells upon infection [26], CATHs are mainly associated with polymorphonuclear neutrophil granulocytes (PMN), where these proteins are abundantly stored preformed inside cytoplasmic granules [31]. The cell type composition is quite different in the milk of the two species; in uninfected sheep milk, macrophages predominate (46 to 84%) and PMN represent only 2–28% of the total cell population. Upon mammary gland infection, PMN increased dramatically, from 50% at an SCC of 200 × 10^3^ cells/mL up to 90% at SCC higher than 3000 × 10^3^ cells/mL. In goats, PMN are already 45–74% of all milk cells in physiological conditions and rise to 71–86% upon infection [15,32]. Therefore, a non-specific SCC increase originating from a reduction of the total milk volume, such as the one occurring before dry-off, translates into a non-specific increase in PMN and consequently also in CATH. Conversely, in sheep, it leads to an increase in other cell types, mainly macrophages, and CATH does not increase. If the SCC increases as a result of an infection, however, the relative increase in PMN against all other cell types leads to a correspondent increase in CATH. Together with CATH produced by mammary epithelial cells in response to microbial stimulation, this provides a specific indication of an inflammatory reaction. Basically, by measuring CATHs, it becomes possible to keep track of the differential changes in somatic cell populations, these proteins being associated specifically with PMNs and with mammary epithelial cells activated by the presence of intramammary pathogens. This explains also the higher specificity of CATH in comparison to SCC in late lactation sheep milk. In goats, the relative PMN increase occurring upon infection is probably too low to offset the physiological level of PMN in healthy milk; CATH, being linked to this cell type, is therefore affected by the same issue. In this scenario, even CATH produced by epithelial cells upon infection might not be enough to compensate the physiological increase in PMN occurring in late lactation goat milk.

This hypothesis is reinforced by the number of CATH-negative and CATH-positive samples in the two species and by their relationship with the SCC. In sheep, with an IMI prevalence of 10.79% according to BC, 25.08% of all milk samples were CATH-positive and had median SCC values above 1700 × 10^3^ cells/mL. When considering that BC on a single milk sample has a theoretical sensitivity of 42.90% in conditions comparable to the ones applied in this study [17], it is plausible that the 25.08% of sheep milk samples positive to CATH and with SCC above 900 × 10^3^ cells/mL (the first three quartiles, i.e., 75% of the distribution) were actually affected by IMI. Accordingly, the test performances of CATH in sheep might even be underestimated when considering the limited sensitivity of the gold standard BC [7,17,18,28]. In goats, on the other hand, IMI prevalence was 15.25% according to BC, but 47.98% of samples were CATH-positive, with median SCC values around 1000 × 10^3^ cells/mL. This also points to a poor specificity of the CATH assay in this small ruminant species.

Looking at ewes, in our previous study the test performances of CATH in mid-lactation animals were already slightly better than SCC [28]. This study demonstrates that the diagnostic advantage of CATH over SCC increases even further in late lactation ewes. As expected, the specificity of both markers decreased, but the steadier performances of CATH resulted in a specificity advantage of over 9 percentage points over SCC (82.98% for CATH versus 73.67% for SCC) at the same sensitivity of 91.80%, exemplified by the higher AUC of over 0.02. The advantage of CATH over SCC in late lactation ewes is particularly relevant since, as clearly pointed out by Koop et al. [19], with all the due considerations linked to the prevalence of the disease, costs associated with false positive and false negative results, as well as species and contagiousness of intramammary pathogens circulating in the flock, higher specificity is preferable over higher sensitivity in mastitis detection.

Another interesting observation is related to the SCC thresholds associated with IMI in sheep milk. From our results, 75% of all milk samples with SCC below 350 × 10^3^ cells/mL were CATH-negative, and 75% of all milk samples above 911 × 10^3^ cells/mL were CATH-positive. This is in line with the recommendation that an SCC of < 500 × 10^3^ or > 1000 × 10^3^ cells/mL should reliably indicate the absence or presence of an IMI, respectively [3,6,16], and suggests that these thresholds might be applicable also in late lactation sheep milk.

Nevertheless, different practical and economical aspects should also be considered. Assessment of the SCC in the field is made possible by a cheap and convenient assay, the CMT, and it has also been implemented in dipstick formats such as the portaSCC. Also, in farms applying dairy herd improvement (DHI) programs, SCC data can be retrospectively recovered and analyzed for lactation trends in each animal or half-udder. On the other hand, CATH could reliably be measured in batches from stored frozen samples with a standard, economical and widespread method as the ELISA. Implementation in the field might also be made possible with dedicated immunoassay formats, including lateral flow or biosensor devices. In sheep, the significant advantage provided by the remarkably higher specificity of CATH might justify its adoption for screening late lactation animals when a more reliable measurement is needed.

## 4. Materials and Methods 

### 4.1. Animals and Milk Samples

The 538 half-udder milk samples included in the study belonged to three sheep flocks (315) and two goat herds (223). The three semi-intensive dairy sheep farms were located in Sardinia (Italy) and all animals belonged to the Sarda breed. All were free of mycoplasmosis and paratuberculosis. Ewes lambed between October and November and lambs suckled from their mothers. The multiparous to primiparous sheep ratio was approximately 4:1. The sheep milk samples included in the study were collected at the end of June, when only one daily milking was already being performed. The two goat farms were located in Lombardy (Italy). One herd was composed only by Alpine goats (60) and one herd only by Saanen goats (163). All were free of mycoplasmosis, brucellosis, paratuberculosis, and caprine arthritis-encephalitis. Goats were housed during winter and grazed freely during the day from June to September. Does kidded between February and March and kids suckled from their mothers. The multiparous to primiparous goat ratio was approximately 3:1. The goat milk samples included in the study were collected at the beginning of November. Milk sampling was done in the context of routine voluntary herd screening programs for mammary gland health improvement and was performed by the respective farm veterinarians according to the standards set by the National Mastitis Council (2019). Before sampling, all animals were clinically examined for udder health. Goat herds were free of clinical mastitis, and very few cases were observed in sheep. For milk collection, teats were carefully cleaned and disinfected using disposable towels embedded with chlorhexidine, foremilk was stripped, and about 40 mL (ewes) or 10 mL (does) of milk was collected aseptically from each half-udder into sterile vials. Samples were brought refrigerated to the diagnostic laboratory (IZS Sardegna for sheep milk and University of Milan for goat milk) where BC and SCC were performed within 24 hours. Aliquots were frozen and delivered in temperature-controlled containers to Porto Conte Ricerche (Alghero, Italy) for ELISA testing. Only samples from clinically healthy udders were included in the study cohort.

### 4.2. Bacteriological Culture (BC) and Somatic Cell Count (SCC)

BC was performed according to the National Mastitis Council recommendations (2017). Ten µl of milk was spread onto blood agar plates (5% defibrinated sheep blood) and incubated aerobically at 37 °C. Plates were examined after 24 h for colony growth. Colonies were counted and evaluated for morphology and presence of hemolysis. When needed, representative colonies were sub-cultured in appropriate solid culture media to obtain pure cultures. Gram-positive cocci were tested by the catalase reaction for differentiation into staphylococci and streptococci. All isolated staphylococci were subjected to the coagulase test for classification into *Staphylococcus aureus* or non-aureus species, and sub-cultured in Baird-Parker or Mannitol salt agar for further confirmation. Samples with fewer than five colonies with the same characteristics were classified as negative, while samples with more than two different colony types were classified as contaminated. The SCC was evaluated with an automated counter (Bentley Somacount 150; Bentley Instruments, Chaska, MN, USA—goat milk, and Fossomatic FC; Foss Electric, HillerØd, Denmark—sheep milk) [33]. 

### 4.3. Pan-Cathelicidin ELISA

CATH abundance was assessed with a sandwich ELISA developed in-house at Porto Conte Ricerche (Alghero, Italy) [34]. The optical density value (OD) measured at 450 nm (OD450) was normalized against six culture-negative sheep or goat milk samples, according to the species on which the test was performed, with < 50,000 cells/mL, which were included in all ELISA plates as a negative control and as a reference for defining the assay threshold. At assay completion, the average OD450 (+3 SD) of negative controls was subtracted from all OD450 values to calculate the normalized OD450 value (NOD450). For facilitating visualization and analysis, the adjusted OD450 value (AOD450) was obtained by adding a correction factor of 0.1 to all NOD450 values.

### 4.4. Statistics

The Shapiro–Wilk normality test revealed that the data followed a non-normal distribution. Therefore, the Mann–Whitney U test was applied for assessing the statistical significance of the differences among result distributions. GraphPad Prism version 5.03 for Windows (GraphPad Software, La Jolla, CA, USA) was used for descriptive statistics (median and IQR), for statistical analysis, and for calculating ROC curves, Se and Sp. Youden’s index J was calculated as ((Se + Sp) – 100), while c* is the value of SCC or AOD associated with J.

## 5. Conclusions

Sheep can be screened for mastitis at the end of lactation, while goats should preferably be tested at peak lactation, as also suggested by others [19]. In late lactation sheep, CATH should be preferred over SCC for its higher specificity, but careful cost/benefit evaluations will have to be made.

## Figures and Tables

**Table 1:** Median and interquartile ranges of the somatic cell count (SCC) and cathelicidin (CATH) Adjusted Optical Density at 450 nm (AOD450) levels in bacteriological culture (BC) positive and BC negative late lactation milk.

Class	*N*	Median (IQR) ^a^ Cells × 10^3^/mL	Median (IQR) CATH AOD450
Sheep, BC negative	281	235.0 (122.5–554.5)	0.0861 (0.0701–0.1071)
Sheep, BC positive	34	1637.0 (842.8–14,422.0)	0.2261 (0.1352–2.275)
Goats, BC negative	189	303.0 (104.0–772.5)	0.1121 (0.0886–0.1501)
Goats, BC positive	34	812.5 (232.3–2397.0)	0.1148 (0.1152–0.2449)

^a^ IQR, interquartile range.

**Table 2:** Test characteristics observed for somatic cell count (SCC) and cathelicidins (CATH) in late lactation sheep and goat milk.

Test.	AUC ^a^ (95% CI) ^b^	c* ^c^	*J* ^d^	Se% (95% CI) ^e^	Sp% (95% CI) ^f^
*Sheep*					
SCC ^g^	0.8829 (0.8380–0.9279)	488.5	66.36	94.12 (80.32–99.28)	72.24 (66.61–77.40)
CATH ^h^	0.9041 (0.8682–0.9399)	0.1206	74.10	91.18 (76.32–98.14)	82.92 (78.00–87.13)
*Goats*					
SCC	0.6693 (0.5811–0.7576)	422.0	29.79	64.71 (46.49–80.25)	65.08 (57.82–71.85)
CATH	0.6623 (0.5633–0.7613)	0.1183	33.61	76.47 (55.83–89.25)	57.14 (49.76–64.30)

^a^ AUC, area under the curve; ^b^ 95% CI, 95% confidence interval; ^c^ c*, optimal cut-point; ^d^ *J*, Youden Index; ^e^ Se, sensitivity; ^f^ Sp, specificity; ^g^ SCC, cells × 10^3^ / mL; ^h^ CATH, AOD450nm.

## Supplementary Materials

The following are available online at https://www.mdpi.com/2076-0817/9/1/37/s1, Table S1: Milk sample data. Table S2: Summary of bacteriological culture (BC), cathelicidin ELISA (CATH), and somatic cell count (SCC) results in late lactation sheep milk. Table S3: Summary of bacteriological culture (BC), cathelicidin ELISA (CATH), and somatic cell count (SCC) results in late lactation goat milk.
