# Supplementary material for: Relationship of Late Lactation Milk Somatic Cell Count and Cathelicidin with Intramammary Infection in Small Ruminants
Source: Pathogens. 2020 Jan 1;9(1):37. doi: 10.3390/pathogens9010037 (PMC7168667; doi:10.3390/pathogens9010037)
Supplement: Supplementary file 1 [file pathogens-09-00037-s001.zip › pathogens-660910-supplementary/supplementary/Supplementary file 2.docx]

**Supplementary Tables**

**Table S2.** Summary of bacteriological culture (BC), cathelicidin ELISA (CATH), and somatic cell count (SCC) results in late lactation sheep milk.

|  |  | BC | | | |
| --- | --- | --- | --- | --- | --- |
|  |  | Pos | | Neg | |
|  |  | 34 | | 281 | |
|  |  | CATH | | | |
|  |  | Pos | Neg | Pos | Neg |
| SCC | Neg | 2 | 0 | 9 | 194 |
|  | Pos | 29 | 3 | 39 | 39 |

CATH positivity threshold: AOD 0.1206; SCC positivity threshold: 488.5 x 10^3^ cells/mL of milk.

**Table S3.** Summary of bacteriological culture (BC), cathelicidin ELISA (CATH), and somatic cell count (SCC) results in late lactation goat milk.

|  |  | BC | | | |
| --- | --- | --- | --- | --- | --- |
|  |  | Pos | | Neg | |
|  |  | 34 | | 189 | |
|  |  | CATH | | | |
|  |  | Pos | Neg | Pos | Neg |
| SCC | Neg | 6 | 6 | 23 | 100 |
|  | Pos | 20 | 2 | 58 | 8 |

CATH positivity threshold: AOD 0.1183; SCC positivity threshold: 422 x 10^3^ cells/mL of milk.
