# Supplementary figures and images for: Relationship of Late Lactation Milk Somatic Cell Count and Cathelicidin with Intramammary Infection in Small Ruminants
Source: Pathogens. 2020 Jan 1;9(1):37. doi: 10.3390/pathogens9010037 (PMC7168667; doi:10.3390/pathogens9010037)

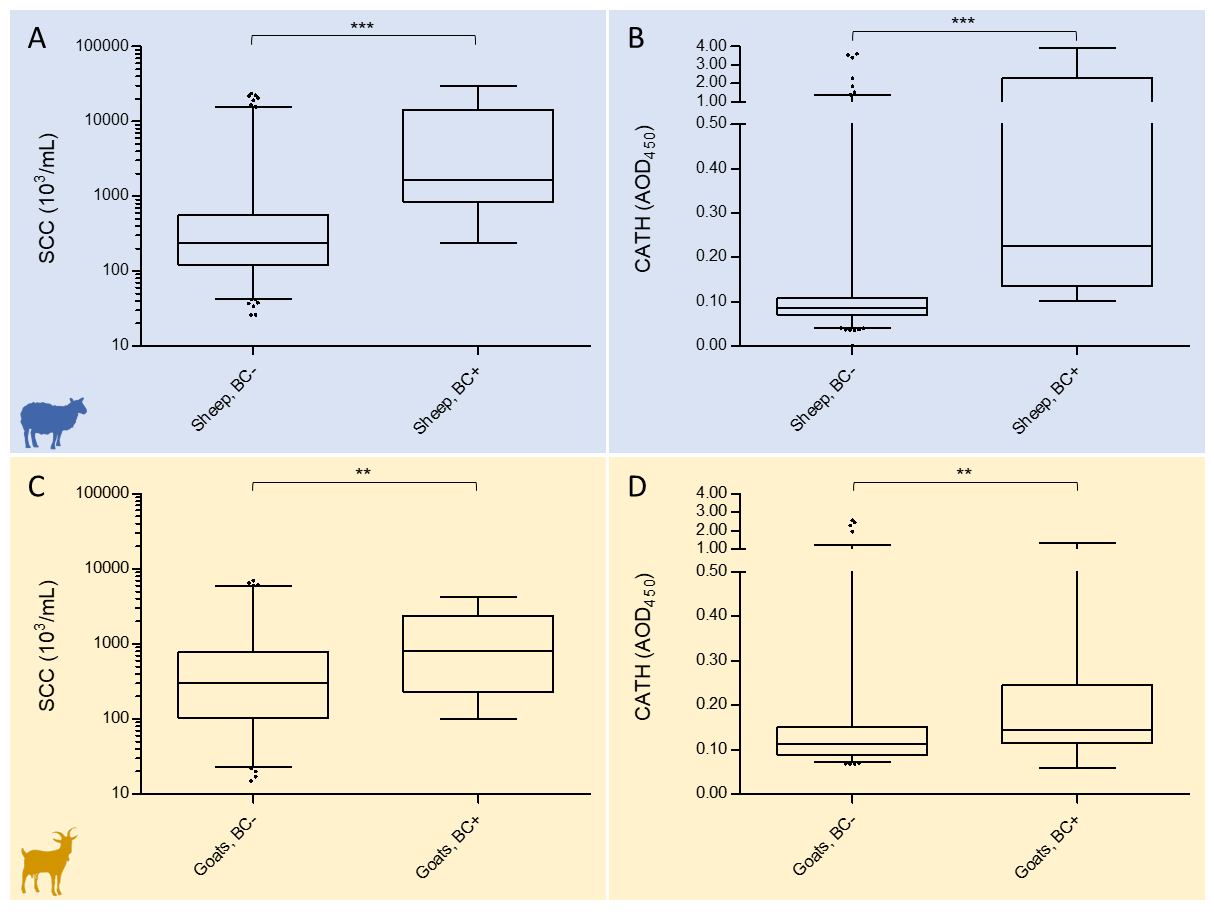

Supplement: Supplementary file 1 [file pathogens-09-00037-s001.zip › pathogens-660910-supplementary/supplementary/Figure 1.tiff]

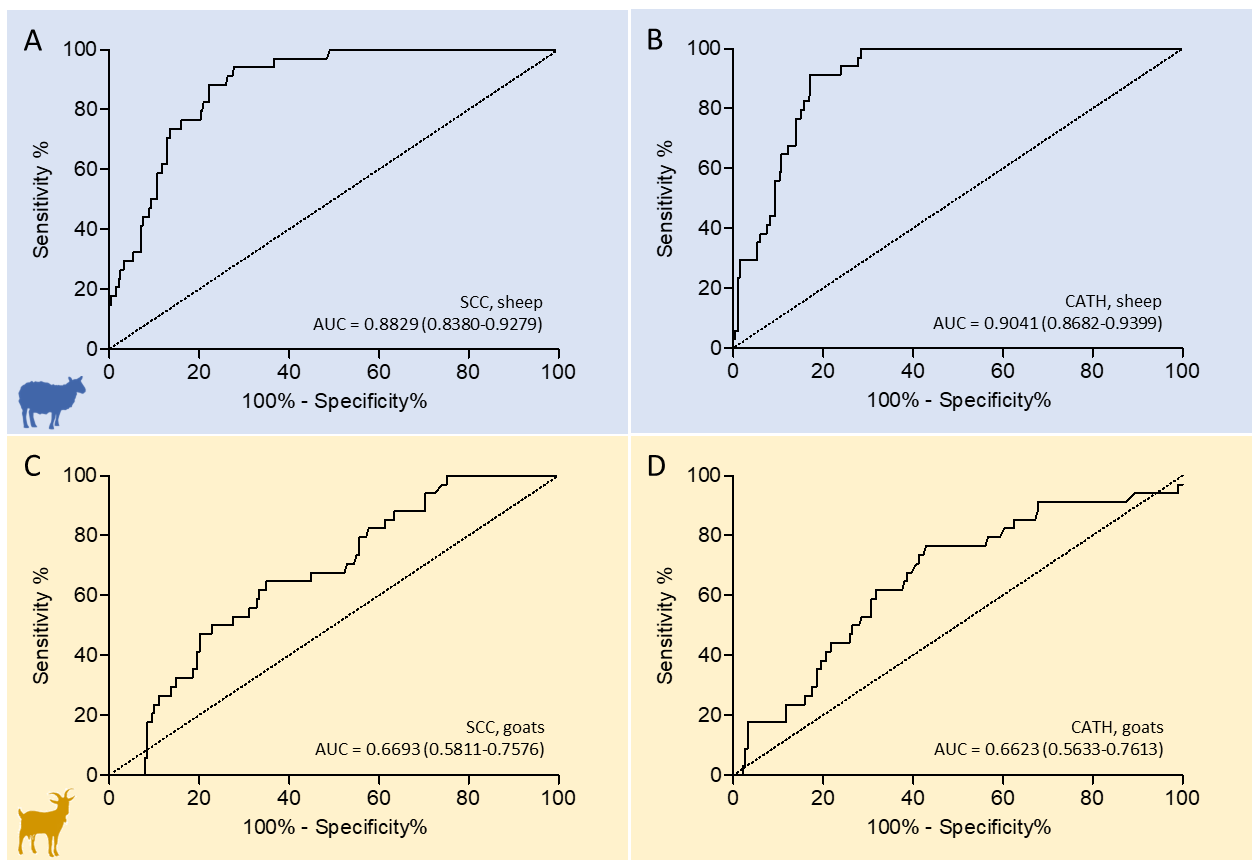

Supplement: Supplementary file 1 [file pathogens-09-00037-s001.zip › pathogens-660910-supplementary/supplementary/Figure 2.tiff]

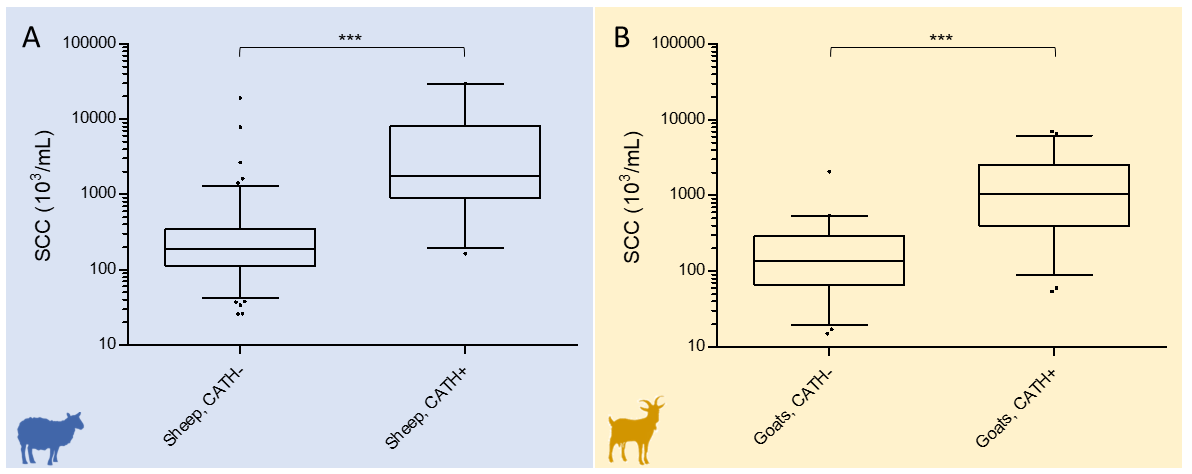

Supplement: Supplementary file 1 [file pathogens-09-00037-s001.zip › pathogens-660910-supplementary/supplementary/Figure 3.tiff]
